# Desvenlafaxine vs SSRI as Lead-in Antidepressants in Intravenous Ketamine Treatment for TRD with Suicidality: A Randomised Controlled Trial Protocol

**DOI:** 10.1192/j.eurpsy.2026.11785

**Published:** 2026-07-31

**Authors:** P. Argitis, M. Demetriou, T. Koukouras, M. Routsi, Z. Chaviaras

**Affiliations:** 1 Psychiatric, General Hospital of Corfu, Corfu, Greece; 2 Psychiatric, General Hospital of Corfu, Corfu, Cyprus; 3 Social Worker, General Hospital of Corfu, Corfu, Greece

## Abstract

**Introduction:**

Intravenous (IV) ketamine has demonstrated rapid antidepressant and anti-suicidal effects in treatment-resistant depression (TRD), yet interindividual variability in response remains significant. One unexplored factor is the impact of concomitant antidepressant medication during ketamine treatment. Desvenlafaxine, a serotonin-norepinephrine reuptake inhibitor (SNRI), may enhance ketamine efficacy through synergistic neurochemical and neuroplastic mechanisms.

Desvenlafaxine may exert such effects via BDNF upregulation and noradrenergic activation, potentially priming glutamatergic pathways for mTOR-mediated synaptic plasticity.

**Objectives:**

To assess whether concomitant initiation of desvenlafaxine during IV ketamine infusions leads to superior clinical outcomes compared to SSRI (sertraline) co-initiation in patients with TRD and active suicidal ideation.

**Methods:**

This is a double-blind, parallel-arm, randomised controlled trial (RCT). A total of 120 adult patients (aged 18–65) diagnosed with TRD (MADRS ≥30) and current suicidal ideation (C-SSRS ≥1) will be randomised to receive:

**Group A:** Desvenlafaxine 50–100 mg/day

**Group B:** Sertraline 50–100 mg/day

Both antidepressants will be initiated **on the same day as the first ketamine infusion**. All patients will undergo 6 IV ketamine infusions (0.5 mg/kg over 40 minutes), administered twice weekly for 3 consecutive weeks.

The **primary outcome** is the **mean change in MADRS** from baseline to day 36.

**Secondary outcomes** include response (≥50% MADRS reduction), remission (MADRS ≤10), change in suicidal ideation (C-SSRS), and time to response. Adverse effects and tolerability will be monitored throughout.

**Results:**

This is a study protocol. It is hypothesised that patients in the desvenlafaxine group will demonstrate greater reductions in depressive symptoms and suicidal ideation, and a more rapid clinical response compared to the SSRI group.

**Table 1.** Study Design and Outcome Measures Table 1. Long description.

Group	Antidepressant Start	Ketamine Dosing	Primary Outcome	Secondary Outcomes
Group A	Desvenlafaxine d1	6 x 0.5 mg/kg IV	ΔMADRS (baseline–d36)	Response, Remission, ΔC-SSRS, Time to response
Group B	Sertraline d1	6 x 0.5 mg/kg IV	ΔMADRS (baseline–d36)	Response, Remission, ΔC-SSRS, Time to response

**Conclusions:**

This study aims to clarify whether concomitant initiation of desvenlafaxine enhances the efficacy of IV ketamine in TRD with suicidality, compared to SSRI initiation. Results may contribute to psychopharmacological strategies that optimise rapid-onset treatments through receptor-level synergy.

If confirmed, these findings may support the implementation of targeted co-initiation protocols that enhance pharmacodynamic alignment between antidepressants and glutamatergic agents.

**Disclosure of Interest:**

None Declared

## Long descriptions

### Table 1. Long description

The table consists of five columns and three rows including the header.

Columns from left to right are: Group, Antidepressant Start, Ketamine Dosing, Primary Outcome, and Secondary Outcomes.

Row 1 (Header): Group, Antidepressant Start, Ketamine Dosing, Primary Outcome, Secondary Outcomes.

Row 2 (Group A):

- Antidepressant Start: Desvenlafaxine d 1.

- Ketamine Dosing: 6 times 0.5 mg/kg I V.

- Primary Outcome: Delta M A D R S (baseline to d 36).

- Secondary Outcomes: Response, Remission, Delta C-S S R S, Time to response.

Row 3 (Group B):

- Antidepressant Start: Sertraline d 1.

- Ketamine Dosing: 6 times 0.5 mg/kg I V.

- Primary Outcome: Delta M A D R S (baseline to d 36).

- Secondary Outcomes: Response, Remission, Delta C-S S R S, Time to response.

Navigate back to Table 1..

